# Effect of Simulated Microgravity on *E. coli* K12 MG1655 Growth and Gene Expression

**DOI:** 10.1371/journal.pone.0057860

**Published:** 2013-03-05

**Authors:** Kotakonda Arunasri, Mohammed Adil, Katari Venu Charan, Chatterjee Suvro, Seerapu Himabindu Reddy, Sisinthy Shivaji

**Affiliations:** 1 Centre for Cellular and Molecular Biology, Hyderabad, India; 2 Vascular Biology Lab, Anna University, Chennai, India; University of Hyderabad, India

## Abstract

This study demonstrates the effects of simulated microgravity on *E. coli* K 12 MG1655 grown on LB medium supplemented with glycerol. Global gene expression analysis indicated that the expressions of hundred genes were significantly altered in simulated microgravity conditions compared to that of normal gravity conditions. Under these conditions genes coding for adaptation to stress are up regulated (*sufE* and *ssrA*) and simultaneously genes coding for membrane transporters (*ompC, exbB, actP, mgtA, cysW* and *nikB*) and carbohydrate catabolic processes (*ldcC, ptsA, rhaD* and *rhaS*) are down regulated. The enhanced growth in simulated gravity conditions may be because of the adequate supply of energy/reducing equivalents and up regulation of genes involved in DNA replication (*srmB*) and repression of the genes encoding for nucleoside metabolism (*dfp, pyrD* and *spoT*). In addition, *E. coli* cultured in LB medium supplemented with glycerol (so as to protect the cells from freezing temperatures) do not exhibit multiple stress responses that are normally observed when cells are exposed to microgravity in LB medium without glycerol.

## Introduction

Microbes have the ability to sense and respond to environmental changes occurring in their vicinity. This adaptability confers on them the capacity to thrive under various extreme environmental niches including microgravity. When exposed to microgravity cells experience reduced gravity resulting in a relative lack of sedimentation, low shear stress and low turbulence [1]. These physical effects of microgravity may influence the growth and also induce other physiological changes. Studies have indicated that bacteria exposed to reduced gravity exhibit up regulation of genes involved in starvation response, acid stress, osmotic stress, oxidative stress, biofilm formation, curli biosynthesis and lipid biosynthesis [1]–[3]. Resistance towards antibiotics and increase in virulence was also reported in different bacteria exposed to microgravity [4]–[6]. The above effects of microgravity on bacteria are possibly dependent on the specific media used for culturing the bacteria [7]–[9].

Functional genomic technologies [10] using *Escherichia coli* strains as a model organism have provided insights in to the effects of microgravity at the genome level. Tucker et al., [11] demonstrated that the expression of genes in *E. coli* under microgravity conditions varied depending on whether the culture was grown on minimal medium or rich medium like Luria-Bertani (LB) broth [11]. Subsequently, Vukanti et al., [1] using DNA microarrays and *E. coli* K 12 identified a number of genes that were significantly altered in expression under microgravity conditions [1]. In two recent studies again in *Escherichia coli* it was clearly demonstrated that microgravity induced expression of multiple stress genes depends on the nutritional status and nutrient resources modulate the responses [12]–[13]. Further, in *Salmonella typhimurium* microgravity decreased the virulence of the bacteria if LB medium was supplemented with phosphate ions [6]. Taken together all these studies substantiate that media composition influences the changes occurring in gene expression under microgravity condition.

In this study, a clinostat that mimics microgravity conditions was used to investigate the effects of microgravity on *E. coli* K12 MG1655 grown in LB medium supplemented with glycerol to monitor the effects on growth and global gene expression. The need for supplementing LB with glycerol was crucial because the incubator in which the bacteria is proposed to be grown at 30°C in space, under microgravity conditions, in a space recovery capsule due to unforeseen reasons may fail and the incubation temperature may decrease to below 0°C. Such unforeseen failures are not very uncommon in space flights. Haga and Saleh [14] analysed 168 satellites that were launched during 1995 to 2009 and recorded a total of 170 failure events which included 62 minor, 105 major and 3 fatal failure events. Thus, it may be essential to culture the bacterium in the presence of glycerol to protect the cultures from any kind of power failure in the spacecraft resulting in freezing temperatures.

The present study differs from the earlier studies in that the LB medium used in the present study is supplemented with glycerol. There is a reasonable chance to assume that the response of the bacterium to microgravity may vary with respect to growth and expression of genes. These assumptions are based on the earlier findings that media components influence the growth and expression of genes of bacteria exposed to microgravity [11]–[13]. In addition it would be interesting to see how glycerol supplementation in the medium would help to overcome the nutrient limiting conditions imposed due to a nutrient depletion zone formed when cells are grown under microgravity conditions [1], [9].

The results of the present study is in accordance with earlier studies that had indicated that *E. coli* exhibits enhanced growth rate and that several genes are differentially expressed when exposed to microgravity [1], [12]–[13]. But, the genes that were differentially regulated were not identical. For instance Vukanti et al., [1] observed that many of the genes that were up regulated were identified as stress-inducible genes and included starvation-inducible genes, genes associated with multiple stress responses, genes involved in biofilm formation, curli biosynthesis and lipid biosynthesis [1]. None of these genes were up regulated in the current study implying that the presence of glycerol in both the control and microgravity exposed cultures nullified the up regulation of stress-inducible genes.

Further, the genes that facilitate the formation of the nutrient depletion zone in cells grown under microgravity conditions such as up regulation of genes coding for nutrient transport and metabolic enzymes and simultaneously down regulation of genes associated with translation apparatus, DNA replication and cell division [1] were not differentially expressed in the presence of glycerol in the medium. Thus implying that glycerol may not favour the formation of a nutrient depletion zone since it could be utilized as a carbon source. In fact the study also indicates that the enhanced growth of *E. coli* under microgravity may be because of adequate supply of energy/reducing equivalents and increase in transcripts for DNA replication.

This study also for the first time assigns the differentially regulated genes to sixteen different functional pathways such as several metabolic pathways (purine, pyrimidine, carbohydrate, amino acid etc.), ABC transporter systems, two component systems etc., by KEGG (Kyoto Encyclopedia of Genes and Genomes). In addition, DAVID (Database for Annotation, Visualization and Integrated Discovery) was used for geneontology annotations and the analysis yielded up regulated genes involved in DNA transcription and regulation of transcription, genes coding for integral membrane proteins, ion binding and DNA binding etc. The 21 down regulated genes were implicated in nitrogen and amine compound biosynthetic process, ion transport, carbohydrate catabolic process and nucleoside metabolic process. DAVID analysis also annotated genes involved in cellular components such as cell wall, cell membrane and organelle membrane and seven hypothetical protein coding genes namely *yfjD, ydcQ, ynfA, ybdJ, yniB, ygaQ* and *hokE* were assigned to various functional processes in response to microgravity conditions in *E. coli*.

## Materials and Methods

### Bacterial Strain and Growth Conditions

In this study, *Escherichia coli* K12 MG1655 was grown in LB broth (containing 10 g peptone, 10 g NaCl and 5 g yeast extract in 1000 ml distilled water) which was supplemented with 10% (v/v) glycerol (hereafter LB) at 30°C. To develop the inoculums, a single colony of *E. coli* K 12 MG1655 from the LB agar plate was inoculated into LB broth and incubated for growth under shaking at 150 rpm and at 30°C. *E. coli* growth was monitored by measuring absorbance at 600 nm. *E. coli* grown to a OD of 0.8 (10^6^ CFU/ml) was used as inoculum for all the experiments unless otherwise mentioned.

### Growth of *E. coli* K12 MG1655 in a Clinostat

A three-dimensional (3D) clinostat is an apparatus that nullifies the effect of gravity, and it has been used to evaluate the effects of microgravity on cells [1], [15]. A 3D clinostat is capable of randomizing motion and thus cancels the uniform gravity influence and as a consequence an object is subjected to weightlessness which is referred commonly as simulated microgravity. The formula for microgravity (g′) is g′ = ω2R/g where g = 9.8 m/s^2^, R = radius from the centre of rotation, ω = constant angular velocity (ω) where angular velocity is equal to angular displacement in radians/time taken (θ/τ). The angular velocity obtained using the clinostat was 2 rads/s. At this angular velocity, the simulated microgravity is 1×10^−3^ [16]. The microgravity in the present study was 1×10^−3^.

To 300 ml of LB medium 3.0 ml of logarithmically growing *E. coli* culture (0.8 OD) was added and 15 ml of this inoculum was distributed into 15 ml separate vessels and grown in clinostat at 30°C. Under these conditions the g was 1×10^−3^ *g*. After every one hour interval one vessel was detached from the instrument and OD_600nm_ of the culture was determined and used to construct the growth curves. *E. coli* culture that was setup as above and grown in an incubator shaker at 30°C under normal gravity conditions served as control. Data of three experiments that were conducted under similar conditions separately was considered in plotting the growth curves.

For simulated microgravity studies, 50 ml of the LB broth was inoculated with 500 µl of logarithmically growing *E. coli* culture and grown in a 50 ml vessel in a clinostat at 30°C. A control was set up as above and grown under normal gravity conditions in an incubator shaker at 30°C. Three separate vessels were maintained under each condition. When the OD_600nm_ reached 0.8, 10 ml of the culture collected from each of these vessels was suspended in 3 volumes of RNA*later* solution (Ambion Inc., USA) in their respective sample bottles and stored at 4°C. From the stored culture 3 ml was used for the isolation of RNA.

### RNA Extraction

Qiagen RNeasy mini-prep kit was used for the extraction of RNA from about 3 ml of *E. coli* culture. The culture was pelleted, suspended in 200 µl Tris-lysozymemixture at room temperature for 5 min and then subjected to lysis with 700 µl of lysis buffer containing 1% β-mercaptoethanol. Lysis was facilitated by vigorous vortexing the suspension of cells for 5 min. Absolute ethanol (500 µl) was added and the mixture was transferred to a mini-spin column placed in a 2 ml centrifuge tube and centrifuged at 13,000 rpm for 30 s. At this stage, 10 µl of DNase prepared in 90 µl of buffer was added to the columns and the columns were kept at room temperature for 10 min. The columns were then washed at 13,000 rpm for 30 s with 500 µl of wash buffers RW1 and purified RNA was collected in 20 µl of RNase-free water after centrifugation at 13,000 rpm for 2 min. Quality of the purified RNA was assessed following gel electrophoresis and the OD 260/280 ratio of the purified RNA ranged from 1.8–2.0. Quantification of the RNA was done using a Nanodrop spectrophotometer (Nano Drop Technologies, USA).

### cDNA Synthesis

RNA (5–10 µg) extracted as above was used for cDNA synthesis using the first strand cDNA synthesis kit from Invitrogen (Invitrogen Bioservices India Pvt. Ltd., Bangalore). Annealing of the primers with the purified RNA was accomplished by incubating the RNA and primer reaction mix at 70°C for 10 min followed by incubation at 25°C for 10 min. cDNA was then synthesized by adding superscript III and incubating the reaction mixture at 25°C for 10 min, 37°C for 60 min, 42°C for 60 min and 70°C for 10 min. The cDNA was fragmented with DNAse 1 (Promega Corporation, Madison, USA) and then labelled with biotin at the 3′ end using the labelling reagent from Affymetrix (CA, USA) and Terminal transferase enzyme (Promega).

### DNA Microarray Analysis

*E.coli* Genome 2.0 gene chip arrays were purchased from Affymetrix. The chip contained the complete genome of four *E. coli* strains (viz., non-pathogenic *E. coli* K12 MG1655, uropathogenic *E. coli* strain CFT073 and enterohemorragic *E. coli* O157:H7 strains EDL 933 and Sakai). The gene chip consists of approximately 10,000 probe sets for the 20,366 genes of all the four strains of *E. coli*. DNA micro array chips were hybridized with the labelled cDNA using the Affymetrix protocol (www.affymetrix.com). Microarray slides were then scanned using Affymetrix 428 Array Scanner and GCOS software to obtain images of the chips and further processed to get intensity cell files for the probe sets. The intensity cell files were then imported normalized for background correction and data analysed using Gene Spring 11.5 software. Genes that exhibited ≥1.5 fold increase or decrease (treated versus control) in expression and P≤0.05 were considered as differentially regulated due to microgravity.

The microarray data was submitted to Gene Expression Omnibus (GEO) web deposit of National Centre for Biotechnology Information (NCBI) with an accession number GSE40648.

### Real Time PCR

The RT-PCR reactions (10 µl) were performed in triplicate with 2.5 pM primer (Table 1) and SYBER Green PCR Master Mix (Applied Biosystems, CA, USA). Template was pre-incubated at 50°C for 2 min, denatured at 95°C for 10 min and subjected to 40 cycles under the following thermal conditions: 95°C (15 s) and 52°C (30 s). Relative expression of genes was calculated by ΔΔC_T_ method which is based on product cycle threshold (C_T_). Expression of 16S rRNA gene was used as an internal standard for RT-PCR. All values reported represent the mean of at least three independent experiments.

**Table 1:** Primers used for Real-Time PCR analysis of a few genes of *Escherichia coli.*

Sl.No.	Gene	Primer	Sequence (5′-3′)
1	*hyaE*	hyaE-rt F	CTTGACGACTGGCTTACG
		hyaE-rt R	GCCACCTGCCATGTATAG
2	*mdtD*	mdt-rt F	TTATCGTCGGGTACTGGTAG
		mdtD -rt R	GAGTTGACCATCCCTTGTAA
3	*srmB*	srmB-rt F	AACATATTGCTGGCGAAA
		srmB-rt R	GAGGGATTGGCAGAAACT
4	*pyrD*	pyrD-rt F	GAAGAATTGATCCAGGTTGC
		pyrD-rt R	TCCCTGAACAAGAGAACGAT
5	*rhaD*	rhaD-rt F	GCGAATGTTTTTGCATCTCT
		rhaD -rt R	CGAGTGTCTGGTGGTATTC
6	*yicL*	yicL-rt F	CGTCGCAGTTTTTGACTATG
		yicL -rt R	AAAAATCAGCAGGCTAATGG
7	*ldcC*	ldcC-rt F	TGTTGATGCCTGGAGAAA
		ldcC-rt R	TCAAAACCGGGGTAATGT
8	*16S*	16S-rtF	GTGCAATATTCCCCACTGCT
		16S-rtR	CGATCCCTAGCTGGTCTGAG

### Assignment of the Differentially Regulated Genes to Functional Pathways by KEGG and DAVID

Genes identified by the microarray analysis were analyzed to identify relevant functional pathways by KEGG (Kyoto Encyclopedia of Genes and Genomes) and DAVID (Database for Annotation, Visualization and Integrated Discovery). A cutoff p value of 0.05 was used for enriched KEGG pathways and geneontology functions by DAVID. Out of 100 genes that were differentially regulated only 57 genes were considered for analysis using KEGG and DAVID. The remaining 43 genes that coded for unknown or intergenic regions were not included in the analysis.

## Results

### Growth of *E. coli* in the Clinostat

**Figure 1:**
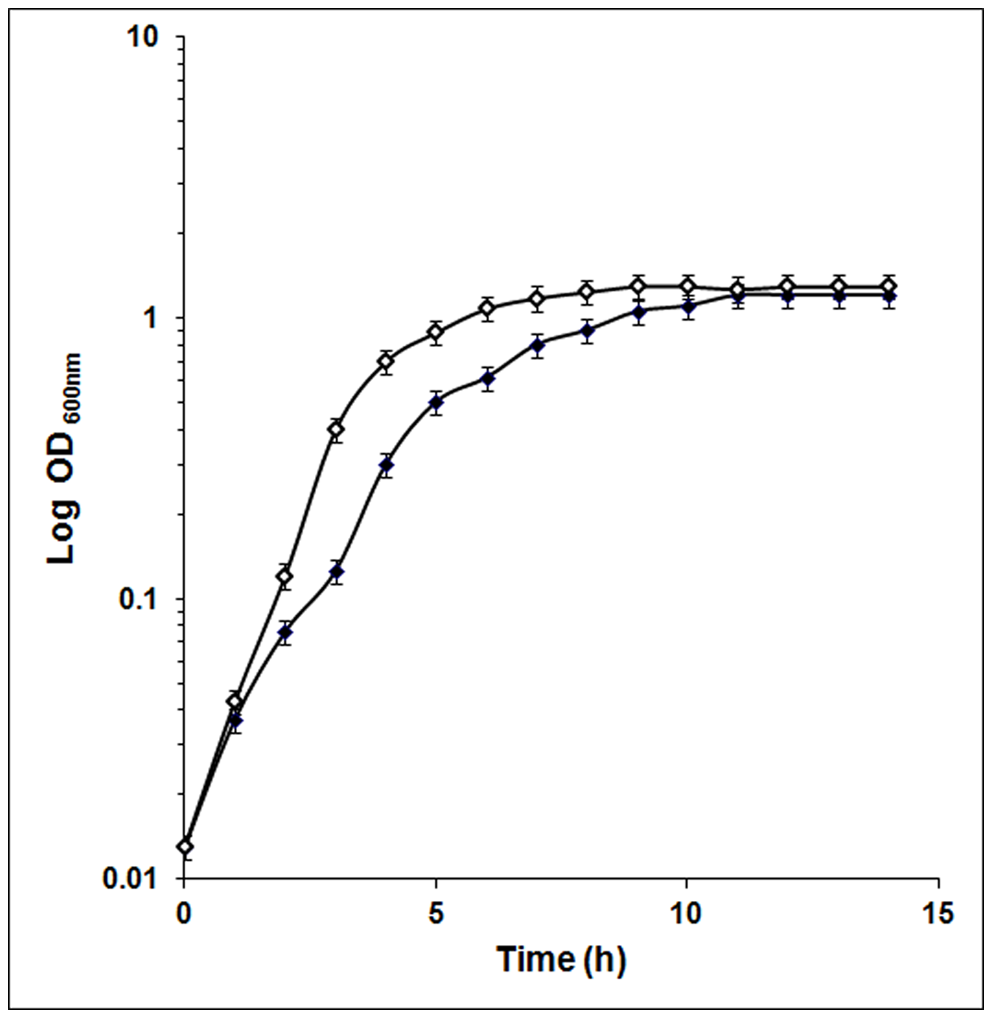
Growth of *E. coli* at 30°C under microgravity conditions in a clinostat (□) and under normal gravity (▪) conditions.

Growth of *E. coli* was monitored under both simulated microgravity conditions and normal gravity conditions. Cultures grown in the clinostat exhibited enhanced growth rate and reached the stationary phase earlier than the culture grown at normal gravity (Fig.1).

### Expression of Genes in *E. coli* Grown in a Clinostat

**Figure 2:**
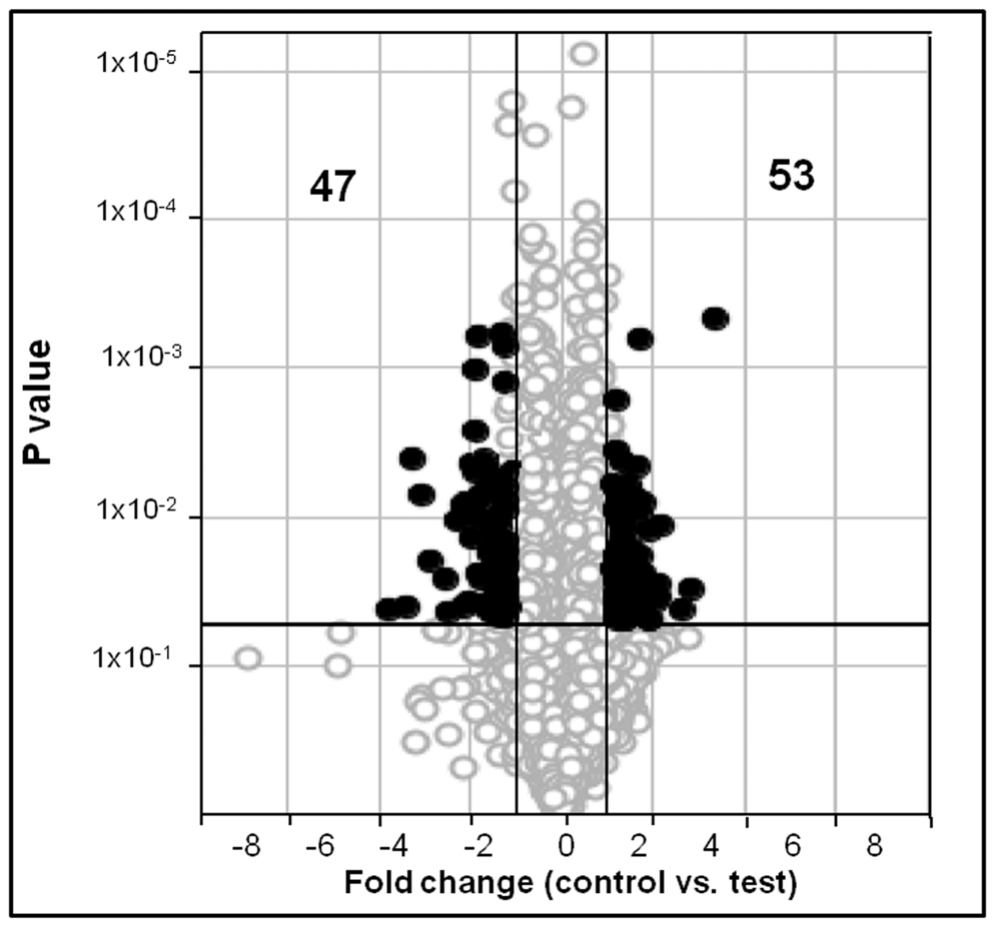
DNA microarray analysis of clinostat-induced gene expression in *E. coli.* The Volcano plot depicts gene expression in *E. coli* culture at 0.8 OD (OD_600 nm_) cultured in the presence of 10% glycerol under microgravity conditions compared to the control. Genes that are represented on the right side of the volcano-axis are up regulated and those that are on left side of the axis are down regulated. Out of the 4377 genes (O) analysed, 53genes were upregulated (•) and 47were down regulated (•).Only those genes that showed more than 1.5 fold change in expression and a P value <0.05 were identified as either up- or down-regulated. The x-axis represents the fold change and the dark vertical lines represent cut-offs at 1.5 fold decrease and increase. The y-axis represents the p-values and the dark horizontal line indicates a p value cut-off of 0.05.

In *E. coli* grown under simulated microgravity conditions hundred genes were differentially expressed with a fold change ≥1.5 (P≤0.05). Three chips were used for control and experimental conditions respectively. Fifty three genes were up regulated while forty seven genes were down regulated under simulated microgravity conditions (Tables 2 and 3 and Fig. 2). The up regulated genes were identified as genes coding for ATP-dependent DNA helicase (*srmB*), multidrug efflux system protein (*mdtD/yegB*), cysteine desulfuration protein involved in oxidative stress (*sufE/ynhA),* periplasmic proteins involved in metal detoxification (*cusF*), chaperone for hydrogenase isoenzyme (*hyaE*), a DNA binding transcriptional regulator (*IlvY*), non coding RNA genes (*ryjA* and *ssrA*), one pseudo gene (*intK*), twelve hypothetical proteins (*ymgG, yaiB, ybdJ, hokE, mokB, ydcQ, ynfA, yniB, ECs3519, ybaM, ydbL* and *ydfO*), thirty one intergenic region genes and one gene coding for an unknown protein (Table 2).

**Table 2:** Microgravity-induced up regulation of genes in *Escherichia coli.*

Probe set ID	Fold change*	Gene	Gene function
1761737_at	1.5	*srmB*	ATP-dependent RNA helicase
1764160_s_at	1.5	*mdtD/yegB*	multidrug efflux system protein
1759467_s_at	1.7	*sufE/ynhA*	cysteine desufuration protein SufE
1761044_at	1.6	*cusF*	periplasmic copper-binding protein
1761315_s_at	1.5	*hyaE*	hydrogenase-1 operon protein HyaE
1768123_s_at	1.6	*ilvY*	DNA-binding transcriptional regulator IlvY
1767538_at	1.5	*intK*	pseudo
1760679_at	2.1	*ryjA*	ncRNA
1762568_s_at	1.7	*ssrA*	misc_RNA/tmRNA
1763277_s_at	1.5	*ymgG*	hypothetical protein
1769259_s_at	1.5	*yaiB*	hypothetical protein
1765637_s_at	1.5	*ybdJ*	hypothetical protein
1764857_s_at	1.8	*hokE*	hypothetical protein
1762609_s_at	1.7	*mokB*	hypothetical protein
1764733_s_at	1.5	*ydcQ*	hypothetical protein
1760864_s_at	1.5	*ynfA*	hypothetical protein
1766918_s_at	1.6	*yniB*	hypothetical protein
1763411_s_at	1.8	*ECs3519*	hypothetical protein
1760935_s_at	1.8	*ybaM*	hypothetical protein
1766171_s_at	1.8	*ydbL*	hypothetical protein
1763909_s_at	1.5	*ydfO*	hypothetical protein
1767707_at	1.7	–	Unknown
1764171_s_at	1.6	–	intergenic region
1767139_s_at	1.5	–	intergenic region
1762045_s_at	1.5	–	intergenic region
1765005_s_at	1.6	–	intergenic region
1768087_s_at	1.6	–	intergenic region
1765131_s_at	1.9	–	intergenic region
1763703_s_at	1.7	–	intergenic region
1761358_s_at	1.8	–	intergenic region
1761890_s_at	1.6	–	intergenic region
1762015_s_at	2.1	–	intergenic region
1761719_s_at	1.7	–	intergenic region
1760269_s_at	1.5	–	intergenic region
1761431_s_at	1.6	–	intergenic region
1767216_s_at	2.1	–	intergenic region
1760296_s_at	1.5	–	intergenic region
1763899_s_at	1.5	–	intergenic region
1759771_s_at	2	–	intergenic region
**Probe set ID**	**Fold change** *	**Gene**	**Gene function**
1765387_s_at	1.8	–	intergenic region
1765961_s_at	1.5	–	intergenic region
1762257_s_at	2	–	intergenic region
1767591_s_at	2	–	intergenic region
1761987_s_at	1.6	–	intergenic region
1765027_s_at	1.8	–	intergenic region
1765126_s_at	1.7	–	intergenic region
1762765_s_at	1.5	–	intergenic region
1767249_s_at	1.6	–	intergenic region
1764126_s_at	2.5	–	intergenic region
1767857_s_at	1.8	–	intergenic region
1766406_s_at	1.8	–	intergenic region
1764615_s_at	1.6	–	intergenic region
1763140_s_at	2	–	intergenic region

* Genes that showed fold change greater than 1.5 (P<0.05).

**Table 3:** Microgravity-induced down regulation of genes in *Escherichia coli.*

Probe set ID	Fold change*	Gene	Gene function
1768937_at	1.6	*ompC*	outer membrane porin protein C
1767282_s_at	1.5	*hisA*	1-(5-phosphoribosyl)-5-[(5-phosphoribosylamino)methylideneamino] imidazole-4-carboxamide isomerase
1766205_x_at	2.4	*hisL*	his operon leader peptide
1763370_s_at	1.5	*insA*	KpLE2 phage-like element; IS1 repressor protein InsA
1763159_s_at	2	*ldcC*	lysine decarboxylase, constitutive
1761146_s_at	1.5	*livG*	leucine/isoleucine/valine transporter ATP-binding subunit
1765703_at	1.9	*aroP*	aromatic amino acid transporter
1767440_s_at	1.5	*cysW*	sulfate/thiosulfate transporter subunit
1759826_s_at	2.7	*cysH*	3′-phosphoadenosine 5′-phosphosulfate reductase
1761464_at	1.6	*nupC*	nucleoside (except guanosine) transporter
1763832_at	1.7	*actP*	acetate transporter
1759775_at	1.6	*ptsA*	fused predicted PTS enzymes: Hpr component/enzyme I component/enzyme IIA component
1764279_s_at	1.5	*mgtA*	magnesium-transporting ATPase MgtA
1766765_s_at	1.5	*nikB*	nickel transporter permease NikB
1768074_s_at	1.5	*gst*	glutathionine S-transferase
1769236_s_at	2.1	*rhaD*	rhamnulose-1-phosphate aldolase
1768302_s_at	1.8	*rhaS*	transcriptional activator RhaS
1764298_s_at	1.5	*ydcR*	putative transcriptional regulator
1759346_s_at	1.6	*hiuH*	transthyretin-like protein precursor
1763816_s_at	1.6	*dfp*	bifunctional phosphopantothenoylcysteine decarboxylase/phosphopantothenate synthase
1766833_s_at	1.6	*spoT*	bifunctional (p)ppGpp synthetase II/guanosine-3′,5′-bis pyrophosphate 3′-pyrophosphohydrolase
1759907_s_at	2	*pyrD*	dihydro-orotate oxidase, FMN-linked
1767020_s_at	1.5	*pldB*	lysophospholipase L2
1767318_s_at	2	*exbB*	biopolymer transport protein ExbB
1763278_at	1.5	*pbl*	pseudo
1761419_s_at	2	*speB*	agmatinase
1766645_s_at	2.1	*yicI*	alpha-xylosidase YicI
1767015_at	1.7	*yfjV*	pseudo
1763267_s_at	1.6	*yeaM*	putative ARAC-type regulatory protein
1759710_at	1.5	*ybeR*	predicted protein
1763523_s_at	1.7	*ypjA*	hypothetical protein
1765691_s_at	1.6	*yfaT*	hypothetical protein
1759640_s_at	1.6	*yfjD*	hypothetical protein
**Probe set ID**	**Fold change** *	***Gene***	**Gene function**
1768572_s_at	1.8	*yehP*	hypothetical protein
1768317_s_at	1.6	*yjbG*	hypothetical protein
1765663_s_at	1.8	*ykiA*	hypothetical protein
1762026_at	1.9	–	Unknown
1768646_s_at	1.6	–	intergenic region
1762447_s_at	1.6	–	intergenic region
1761029_s_at	1.5	–	intergenic region
1760120_s_at	1.6	–	intergenic region
1765833_s_at	1.7	–	intergenic region
1766353_s_at	1.6	–	intergenic region
1762591_s_at	3.7	–	intergenic region
1763867_s_at	1.5	–	intergenic region
1766044_s_at	2.4	–	intergenic region
1767764_s_at	2.2	–	intergenic region

* Genes that showed fold change greater than 1.5 (P<0.05).

The genes that were down regulated included a gene coding for outer membrane porin protein C (*ompC*), *his* operon genes (*hisL, hisA*), insertion element repressor protein (*insA*), lysine decarboxylase (*ldcC*), transporter proteins (*actP, aroP, exbB, livG, nupC* and *cysW*), 3′-phosphoadenosine 5′-phosphosulphate reductase (*cysH,*), metal transport proteins (*mgtA* and *nikB*), fused predicted PTS system enzyme (*ptsA*), glutathionine S-transferase (*gst*), rhamulose-1-phosphate aldolase (rhaD), transcriptional activator (*rhaS*), putative transcriptional regulatory protein (*ydcR* and *yeaM*), transthyretin-like protein (*hiuH*), a bifunctional synthase (*dfp),* bifunctional (p)ppGpp synthetase II/guanosine-3′,5′-bis pyrophosphate 3,-pyrophosphohydrolase (*spoT*), dihydro-orotate oxidase (*pyrD*), lysophospholipase L2 (*pldB*), agmatinase (*speB*) and xylosidase (*yicL*). The other down regulated genes include two pseudo genes (*pbl, yfjV*), seven hypothetical protein coding genes (*ybeR, ypjA, yfaT, yfjD, yehP, yjbG* and *ykiA*), one unknown gene and ten intergenic region coding genes (Table 3).

### Validation of the Expression of Genes by RT-PCR

**Figure 3:**
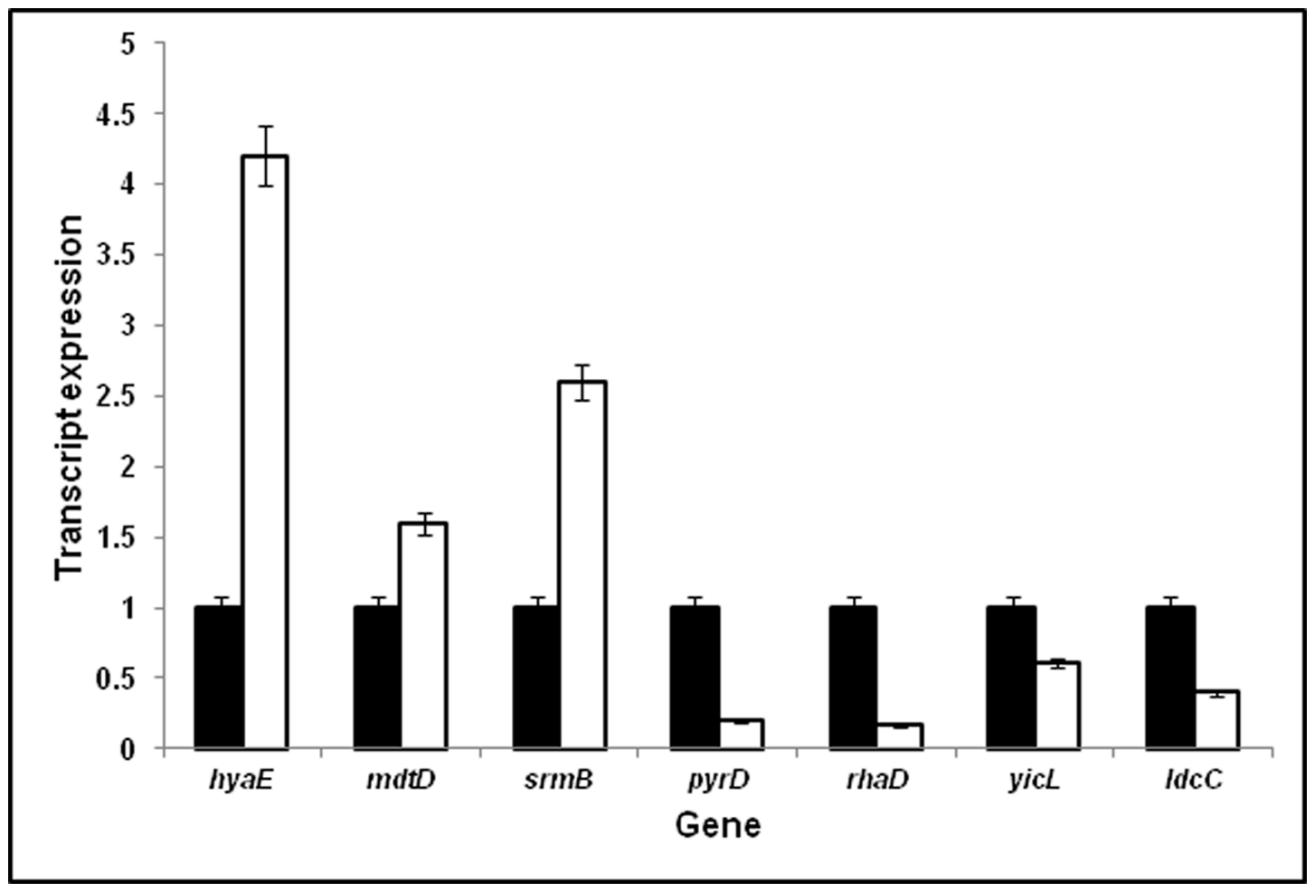
Effect of microgravity on the expression of genes *hyaE, mdtD, srmB, pyrD, rhaD, yicL* and *ldcC* using RNA from *E. coli* grown in a clinostat (▪)and compared with *E. coli* grown in normal gravity conditions (□). The OD of the the cultures was 0.8 (OD_600 nm_) The P values were *pyrD, rhaD* (P<0.001), *yicL* (p<0.05), *srmB, hyaE, mdtD* and *ldcC* (P<0.1) respectively.

RT-PCR was carried out on seven genes (*hyaE, mdtD, srmB, pyrD, rhaD, yicL* and *ldcC*) and the results indicated that, *pyrD* and *rhaD* (P<0.001), *yicL,* (P<0.05), *hyaE, ldcC, mdtD* and *srmB* (P<0.1) were either significantly up regulated or down regulated in accordance with the DNA microarray results (Tables 2 and 3) thus validating the DNA microarray results in *E. coli* exposed to simulated microgravity (Fig. 3). The expression of the genes was calculated based on the product Cycle threshold (C_T_) value. The data was analysed using ANOVA (Prism 3.0 software).

### Assignment of the Differentially Regulated Genes to Functional Pathways by KEGG and DAVID

Out of 100 genes that were differentially regulated only 57 genes were considered for analysis using KEGG and DAVID. The remaining 43 genes that coded for 2 unknown and 41 Intergenic regions were not included in the analysis. Out of 57 genes that were differentially regulated only 14 down regulated and one up regulated gene could be associated with a particular pathway when analysed by KEGG pathways. Hyperlinks have been provided to these genes. These fifteen genes belonged to sixteen different pathways like metabolic pathways (*cysH, dfp, hisA, hiuH, ldcC, pyrD* and *speB),* the two-component system (*cusF* and *ompC*), purine metabolism (hiuH *and spoT*), microbial metabolism in diverse environments (*cysH,* and *hiuH*), biosynthesis of secondary metabolites (*hisA* and *ldcC*), ABC transporters (*cysW* and *nikB*), glycerophospholipid metabolism (*pldB*), phosphotransferase system (*ptsA*), pantothenate and CoA biosynthesis (*dfp*), pyrimidine metabolism (*pyrD*), fructose and mannose metabolism (*rhaD*), pentose and glucuronate interconversion (*rhaD*), arginine and proline metabolism (*speB*), histidine metabolism (*hisA*), lysine degradation (*ldcC*) and sulfur metabolism (*cysH*). The KEGG pathway analysis also indicated that certain genes like *cysH, hisA, hiuH, ldcC, pyrD, speB,* and *rhaD* were associated with more than one pathway.

**Figure 4:**
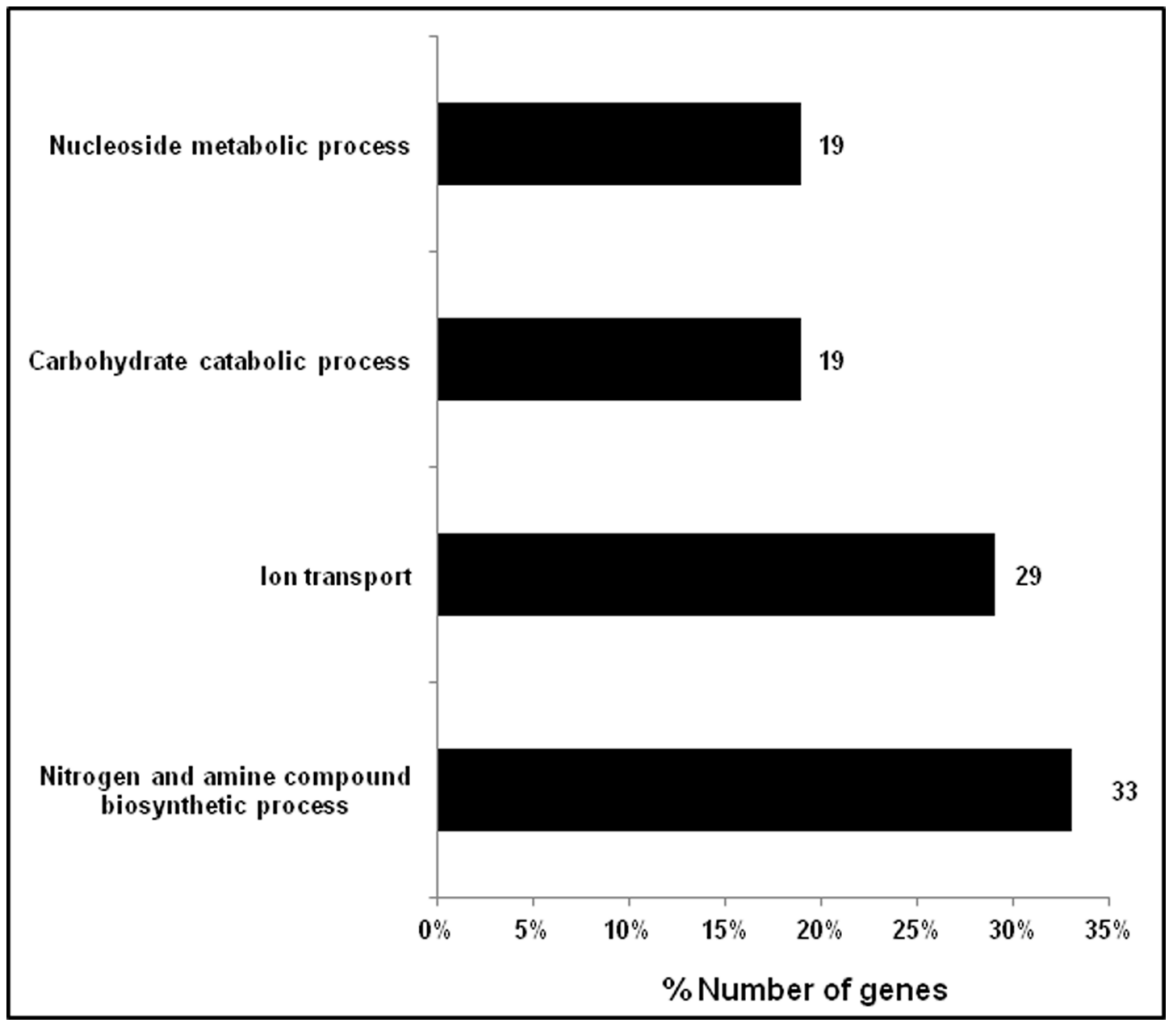
Distribution of down regulated genes (%) based on biological process classification reported by Gene ontology term functional categories using DAVID version 2.0 software.

DAVID was also used for geneontology annotations and term enrichments for various biological processes, molecular functioning and cellular components. Out of 57 genes that were differentially regulated and analysed by DAVID only 21 down regulated and 9 up regulated genes yielded term enrichments. Hyperlinks have been provided to these genes. The analysis yielded that the 9 upregulated genes were involved in two biological processes namely DNA transcription and regulation of transcription (*ilvY* and *ydcQ*), one cellular component which included a number of genes coding for integral membrane proteins (*hokE, mdtD, ynfA, ybdJ* and *yniB*) and three molecular functioning terms including ion binding (*cusF* and *ygaQ*) and DNA binding (*ilvY* and *ydcQ*). The 21 down regulated genes were implicated in four biological processes (Fig. 4) such as genes involved in nitrogen and amine compound biosynthetic process (*hisA, speB, aroP, pyrD, livG, mgtA* and *cysH*), ion transport (*exbB, actP, mgtA, ompC, cysW* and *nikB*), carbohydrate catabolic process (*rhaS, ldcC, ptsA* and *rhaD*) and nucleoside metabolic process (*dfp, pyrD, spoT* and *nupC*). DAVID analysis also annotated genes involved in cellular components such as cell wall, cell membrane and organelle membrane (*aroP, exbB, actP, pyrD, pldB, mgtA, nikB, nupC, ompC, cysW* and *yfjD*). Similarly GO terms enriched for molecular functioning included genes coding for proteins involved in metal-ion binding, cation-binding and ion-binding (*speB, actP, dfp, spoT, mgtA, ptsA, nikB* and *rhaD)*.

## Discussion

Bacteria under reduced gravity conditions exhibit a number of distinct physiological changes such as changes in growth characteristics, increase in resistance to acidic, osmotic, and thermal stress [2], [17], greater tolerance to ethanol exposure [18], enhanced attachment abilities [19], [20], increase in virulence, ability to use substrates more effectively [21] and have altered gene expression [22]. Such studies are best done in a spacecraft in orbit. But due to logistic reasons and practical limitations, studies conducted in ground-based systems that mimic microgravity conditions like using a clinostat, could help to generate data and provide clues to important effects of microgravity on life systems [23]–[25]. A clinostat creates microgravity conditions characterized by reduced sedimentation, low shear and low turbulence as in space flight [25], [26]. Thus studies have used clinostat as a device to subject microbes to reduced gravity conditions and monitor the effects on the growth, physiology and expression of genes in bacteria [27], [28].

Earlier studies demonstrated that *E. coli* under simulated microgravity conditions exhibits decreased lag phase and an increase in exponential phase compared to normal gravity [1], [11], [29], [30]. Our study on growth of *E. coli* on LB medium in a clinostat also showed increased growth rate compared to normal gravity conditions. On the contrary Bouloc and D’Ari [31] showed no difference in growth of *E. coli* in the orbiting Biocosmos 2044 satellite compared to its ground based controls. Studies have indicated that the effects of reduced gravity on bacterial viability and growth is dependent on whether the bacteria were cultured in rich or dilute media. In *S. aureus* growth characteristics were similar in LB under reduced and normal gravity conditions but in 1/10 LB, the growth of *S. aureus* was significantly increased under microgravity [13]. The authors have also suggested that the physiological responses to microgravity conditions vary with growth medium and growth phase. However, recent studies by Berry et al [32] on the effect of hypobaric pressures similar to those of the surface of mars (0.69 kPa global average) on the growth of *E.coli* revealed slight reduction in growth.

In the present study a fold change of ≥1.5 was considered to analyse the data on differential gene expression in simulated microgravity conditions. Several other studies also used a fold change ≥1.5 as a cut-off for the analysis of differential gene expression [33], [34]. In this study using a fold change ≥1.5 cut-off a total of 100 genes were differentially regulated in *E. coli* under simulated microgravity conditions, out of which fifty three were upregulated and forty seven down regulated compared to normal gravity grown culture (Tables 2 and 3).

Wilson et al. [22] were the first to monitor global gene expression in *Salmonella* in response to microgravity. A total of 163 genes were differentially regulated by microgravity. These differentially regulated genes included genes coding for transcriptional regulators, virulence factors, lipopolysaccharide biosynthetic enzymes, iron-utilization enzymes and several genes with no homology to other genes in the current databases. Subsequent studies on *Salmonella* also led to the discovery of novel virulence mechanisms involving a ferric uptake regulator under reduced gravity conditions [3], [6], [22]. Recent studies also revealed that *P. aeruginosa* responded to spaceflight conditions through differential regulation of 167 genes and 28 proteins, with Hfq as a global transcriptional regulator [35]. Vukanti et al. [1] using *E. coli* K12 demonstrated that 430 genes were significantly altered in expression under modeled reduced gravity conditions. Up regulated genes included starvation inducible genes (*csiD, cspD, ygaF, gabDTP, ygiG, fliY* and *cysK*), genes associated with multiple stress responses (*asr, yhiW, yehZYW, katE* and *btuDE*), genes involved in biofilm formation (*lldR, lamB, yneA, fadB* and *ydeY*), curli biosynthesis (*csgDEF*), and lipid biosynthesis (*yfbEFG*). None of these genes were up regulated in the current study. The only difference between the present study and that of Vukanti et al. [1] being that *E. coli* K12 MG1655 in the present study was grown in LB containing glycerol. Therefore the effect of glycerol on gene expression was studied (GEO accession number is GSE34275). It was observed that in *E. coli* K12 MG1655 grown in the presence of glycerol 103 genes were up regulated whereas 209 were down regulated (Supplementary Tables S1 and S2). The results also indicated that the presence of glycerol in both the control and microgravity exposed cultures nullified the up regulation of starvation induced genes (*cbp*A, *usp*B *and wrb*A), genes associated with multiple stress responses (*hdeA, hdeB, hdeD, gadA, gadB, gadC, gadW, cadB, dhaL, dhaK, ompW, tar* and *tnaL*), genes involved in curli biosynthesis (*fliC, fimA*), and lipid biosynthesis (*ybjP*) as observed by Vukanti et al. [1]. The mechanism or the reason for the difference observed still remains unknown. A few of the differentially regulated genes were also validated for their expression by RT-PCR and found to be consistent with the DNA microarray results (Tables 2 and 3 and Fig. 2).

Earlier studies demonstrated that cells grown under microgravity conditions develop a nutrient depletion zone around the cells [24]. As a consequence, genes coding for nutrient transport and salvage systems, multiple stress resistance and metabolic enzymes are up regulated and simultaneously down regulation of genes associated with translation apparatus, DNA replication and cell division were observed [1]. However, surprisingly in the present study the above genes were not differentially expressed implying that the presence of glycerol in the medium may not favour the formation of a nutrient depletion zone. In fact this may be so because glycerol can enter the cell through simple diffusion and thus get utilized as a carbon source and generate acidic metabolic products. This may be the reason as to why *E. coli* grown in the presence of glycerol exhibits up regulation of genes related to acid stress (Supplementary Table S1and S2). Recently, Vukanti and Leff [12] also demonstrated that general stress response of the bacteria grown under microgravity can be reversed by supplementing a nutrient rich medium.

Comparison of genes identified in the present study to genes reported in previous studies [1] revealed that only a few differentially expressed genes were common. These genes include *hyaE, livG* and *pyrD*. Gene *hyaE* encodes for HyaE protein of hydrogenase-1 operon which comprises six genes *hyaABCDEF*. In Vukanti et al. [1] all the six genes of the operon were up regulated whereas in the present study only *hyaE* was upregulated. *pyrD* which encodes dihydroorotate dehydrogenase and involved in pyrimidine biosynthesis is down regulated in both the studies. In addition, in the paper of Vukanti et al. [1] few other genes of the *pyr* operon (*pyrF, carA, pyrB, pyrG, pyrC* and *carB*) were also down regulated. Gene *livG* that belongs to *livFGHMK* operon that is involved in high-affinity branched-chain amino acid transport system is down regulated. While *ilvGMEDA* operon an amino acid biosynthetic operon required for the synthesis of the branched chain aminoacids isoleucine, leucine and valine is down regulated in previous studies [1].

In the present study *cysW* involved in sulfate/thiosulfate transport, *cysH* involved in cystein biosysnthesis and gene *actP* that is involved in acetate transport are down regulated, so as to possibly maintain pH homeostasis in the acidified media conditions. This is in contrast to the previous observations that genes pertaining to acetate metabolism *acs* and *actP* and *cysAV* involved in sulfate/thiosulfate transport as well as genes involved in cysteine biosynthesis *cysCDIJKN* were upregulated [1], to overcome oxidative stress due to microgravity conditions [1].

In the present study 100 genes were either up or down regulated when *E. coli* was cultured under microgravity conditions. Analysis based on the DAVID geneontology (GO) term enrichments included the following up regulated genes belonging to DNA transcription (*srmB*) and those involved in regulation of transcription (*ilVY* and *ydcQ*). Gene *srmB* encodes for DNA helicase. Upregulation of helicases is interesting as these plays a vital role in DNA repair, recombination and RNA transcription [36]. At the same time DAVID analysis also revealed enrichment of GO terms such as nucleoside metabolic process for the down regulated genes *dfp, pyrD, spoT.* Gene *dfp* is an essential gene required for DNA synthesis [37] and gene *pyrD* involved in pyrimidine biosynthesis [38] are down regulated. Gene *ydcR* that belongs to one of the transcriptional regulator family GntR is also repressed. The low abundance of these genes may be because of the active growth of *E. coli* culture under microgravity conditions. The *spoT* gene which codes for ppGpp in response to nutrients limitation [39], [40] is also repressed implying that cells are not in a nutrients limiting condition in the clinostat. Based on DAVID analysis, genes involved in carbohydrate catabolic process (*rhaS, rhaD, ldcC* and *ptsA*) are also down regulated suggesting that energy/reducing equivalents are adequately present. Gene *ilvY* is involved in the transcriptional regulation of the *ilvA* gene of the *ilvGMEDA* operon encoding for biosynthesis of branched chain aminoacids leucine, isoleucine and valine [41] is up regulated. It is also observed that DAVID analysis enriched GO terms nitrogen compound and amine biosynthetic processes for down regulated *genes hisA, speB, aroP, pyrD, livG, mgtG* and *cysH*. These results imply that cells growing under microgravity conditions are not deprived of nitrogenous compounds.

The other up regulated genes enriched include those coding for cation-binding (*cusF* and *ygaQ*) and DNA-binding (*ydcQ)*. Tucker et al., [1] reported down regulation of *cusF* when *E. coli* was grown in MOPS medium under simulated microgravity conditions. *cusF* is a periplasmic chaperone and exports copper and silver ions both from cytoplasm and periplasm to the external environment to maintain cell homeostasis. Enrichment of DAVID GO terms for ion transport, metal ion transport and cation transport for the down regulated genes (*ompC, exbB, actP, mgtA, cysW* and *nikB*) is also indicated. These transport systems assist in uptake of essential nutrients, regulate the flow of physiologically relevant chemicals, and release substances such as signalling molecules. Gene *ompC* coding for major outer membrane protein functions as a porin, allows free diffusion of small hydrophilic molecules across the membrane [42] is down regulated. Apart from these ion transporters, detoxification genes like *gst* encoding for glutathione transferase that protects cells by detoxification of metals Hg^2+^ and Cd^2+^ [43] is also down regulated may be because the cells are in an environment free of metal ions.

Based on DAVID enrichment terms the up regulation of genes integral to membrane include *mdtD* along with hypothetical protein coding genes *hokE, ynfA, ybdJ* and *yniB*. Gene *mdtD* that encodes for multidrug efflux system [44] may indicate acquisition of drug resistance by *E. coli* cultured under microgravity conditions. This observation is in accordance with the observations of Leys et al. [45] that bacteria growing in space require greater concentrations of various antibiotics to inhibit their growth. However, Tucker et al. [11] did not observe any significant change in the antibiotic resistance in *E. coli* exposed to microgravity. The up regulation of *sufE* implies that in the prevailing conditions iron was limiting since *suf* genes are induced under iron limitation conditions [46]. Up regulation of gene *ssrA* that encodes for small RNA, is required for adaptation to environmental changes and growth under stress conditions [47] could be an advantage to the growth of the bacterium under microgravity conditions.

On the whole in this study a number of genes that were not reported earlier when *E. coli* is grown on LB media under microgravity conditions appeared when 10% v/v glycerol is additionally supplemented to LB medium. The up regulated genes comprised those that are related to transcription and integral to membrane. Among the down regulated genes, those involved in nitrogen, nucleoside and carbohydrate metabolic process and membrane related transporters were present. We did not observe elevation of multiple stress genes in response to nutrient depletion zone in our study, may be because of the reversal of this stress with glycerol in the medium. Using DAVID seven hypothetical protein coding genes namely *yfjD, ydcQ, ynfA, ybdJ, yniB, ygaQ* and *hokE* were assigned to various functional processes in response to microgravity conditions in *E. coli*. A number of hypothetical and Intergenic region genes were also differentially expressed.

### Conclusions

The results imply that *E. coli* in the presence of glycerol under simulated microgravity conditions grows better than compared to the normal gravity control. The enhanced growth may be because of adequate supply of energy/reducing equivalents and increase in transcripts for DNA replication. Further, glycerol supplementation in the medium helps in overcoming multiple stressors exerted in microgravity conditions due to nutrient limitations.

## Supporting Information

Table S1 **Glycerol-induced up regulation of genes in** ***Escherichia coli.*** DNA microarray analysis of *E. coli* grown in the presence of 10% glycerol showed up regulation of 103 genes with a fold change >1.5 (P<0.05).
(DOCX)

Table S2 **Glycerol-induced down regulation of genes in** ***Escherichia coli*** **.** DNA microarray analysis of *E. coli* grown in the presence of 10% glycerol showed down regulation of 209 genes with a fold change >1.5 (P<0.05).
(DOCX)

## References

[1] Vukanti R, Mintz E, Leff L (2008) Changes in gene expression of *E. coli* under conditions of modelled reduced gravity. Microgravity Sci Technol 20: 41–57.

[2] Nickerson CA, Ott CM, Mister SJ, Orrow BJ, Burns-Keliher L, et al (2000) Microgravity as a novel environmental signal affecting *Salmonella enterica s*erovar Typhimurium virulence. Infect Immun 68: 3147–3152. 10816456 10.1128/iai.68.6.3147-3152.2000 PMC97548

[3] Wilson JW, Ott CM, Bentrup KH, Ramamurthy R, Quicka L, et al (2007) Space flight alters bacterial gene expression and virulence and reveals a role for global regulator Hfq. Proc Natl Acad Sci 104: 16299–16304. 17901201 10.1073/pnas.0707155104 PMC2042201

[4] Rosenzweig JA, Abogunde O, Thomas K, Lawal A, Y-Uyen Nguyen, et al (2010) Spaceflight and modeled microgravity effects on microbial growth and virulence. Appl Microbiol Biotechnol 85: 885–891. 19847423 10.1007/s00253-009-2237-8 PMC2804794

[5] Chopra VA, Fadl AA, Sha J, Chopra S, Galindo CL, et al (2006) Alterations in the virulence potential of enteric pathogens and bacterial–host cell interactions under simulated microgravity conditions. J Toxicol Environ Health A 69: 1345–1370. 16760141 10.1080/15287390500361792

[6] Wilson JW, Ott CM, Quick L, Davis R, Höner zu Bentrup K (2008) Media ion composition controls regulatory and virulence response of *Salmonella* in spaceflight.PLoS ONE. 3: e3923. 10.1371/journal.pone.0003923 PMC2592540 19079590

[7] Baker PW, Leff LG (2004) The effect of simulated microgravity on bacteria from the Mir space station. Microgravity Sci Technol 15: 35–41. 15773020 10.1007/BF02870950

[8] Baker PW, Leff LG (2006) Mir space station bacteria responses to modeled reduced gravity under starvation conditions. Adv Space Res 38: 1152–1158.

[9] Benoit MR, Klaus DM (2007) Microgravity, bacteria and the influence of motility. Adv Space Res 39: 1225–1232.

[10] Simon C, Daniel R (2011) Metagenomic analyses; Past anad future trends. Appl Environ Microbiol 77: 1153–1161. 21169428 10.1128/AEM.02345-10 PMC3067235

[11] Tucker DL, Ott CM, Huff S, Fofanov Y, Pierson DL, et al (2007) Characterization of *Escherichia coli* MG1655 grown in a low-shear modeled microgravity environment. BMC Microbiol 7: 15. 17343762 10.1186/1471-2180-7-15 PMC1852313

[12] Vukanti R, Leff LG (2012) Expression of multiple stress response genes by *Escherichia coli* under modelled reduced gravity. Microgravity Sci Technol 24: 267–279.

[13] Vukanti R, Model MA, Leff LG (2012) Effect of modelled reduced gravity conditions on bacterial morphology and physiology. BMC Microbiol 12: 4. 22239851 10.1186/1471-2180-12-4 PMC3274431

[14] Haga RA, Saleh JH (2011) “Epidemiology of satellite anomalies and failures: A subsystem-centr*ic approach,”* *Aerospace Conference, 2011 IEEE*, 5–12.

[15] Gruener R, Roberts R, Reitstetter R (1994) Reduced receptor aggregation and altered cytoskeleton in cultured myocytes after space-flight. Biol Sci Space 8: 79–93. 11542735 10.2187/bss.8.79

[16] Huijser RH (2000) Desktop RPM: new small size microgravity simulator for the bioscience laboratory. FS-MG-0017, Fokker Space.

[17] Wilson JW, Ott CM, Ramamurthy R, Porwollik S, McClelland M, et al (2002) Low-Shear modeled microgravity alters the *Salmonella enterica* serovar Typhimurium stress response in an RpoS-independent manner. Appl Environ Microbiol 68: 5408–5416. 12406731 10.1128/AEM.68.11.5408-5416.2002 PMC129924

[18] Gao Q, Fang A, Pierson DL, Mishra SK, Demain AL (2001) Shear stress enhances microcin B17 production in a rotating wall bioreactor, but ethanol stress does not. Appl Microbiol Biotechnol 56: 384–387. 11549006 10.1007/s002530100610

[19] Baker PW, Leff LG (2005) Attachment to stainless steel by Mir Space Station bacteria growing under modeled reduced gravity at varying nutrient concentrations. Biofilms 2: 1–7.

[20] McLean RJ, Cassanto JM, Barnes MB, Koo JH (2001) Bacterial biofilm formation under microgravity conditions. FEMS Microbiol Lett 195: 115–119. 11179638 10.1111/j.1574-6968.2001.tb10507.x

[21] Brown RB, Klaus D, Todd P (2002) Effects of space flight, clinorotation and centrifugation on the substrate utilization efficiency of *E. coli* . Microgravity Sci Technol 13: 24–29. 12521048 10.1007/BF02881678

[22] Wilson JW, Ramamurthy R, Porwollik S, McClelland M, Hammond T, et al (2002) Microarray analysis identifies *Salmonella* genes belonging to the low-shear modeled microgravity regulon. Proc Natl Acad Sci 99: 13807–13812. 12370447 10.1073/pnas.212387899 PMC129779

[23] Gao H, Ayyaswamy PS, Ducheyne P (1997) Dynamics of a microcarrier particle in the simulated microgravity environment of a rotating wall vessel. Microgravity Sci Technol 10: 154–165. 11543416

[24] Hammond TG, Hammond JM (2001) Optimized suspension culture: the rotating-wall vessel. Am J Physiol Renal Physiol 281: F12–25. 11399642 10.1152/ajprenal.2001.281.1.F12

[25] Nickerson CA, Ott CM, Wilson JW, Ramamurthy R, LeBlanc CL, et al (2003) Lowshear modeled microgravity: a global environmental regulatory signal affecting bacterial gene expression, physiology, and pathogenesis. J Microbiol Methods 54: 1–11. 12732416 10.1016/s0167-7012(03)00018-6

[26] Nickerson CA, Ott CM, Wilson JW, Ramamurthy R, Pierson DL (2004) Microbial responses to microgravity and other low-shear environments. Microbiol Mol Biol Rev 68: 345–361. 15187188 10.1128/MMBR.68.2.345-361.2004 PMC419922

[27] Yamazaki T, Yoshimoto M, Nishiyama Y, Okubo Y, Makimura K (2012) Phenotypic characterization of *Aspergillus niger* and *Candida albicans* grown under simulated microgravity using a three-dimensional clinostat. Microbiol Immunol 56: 441–446. 22537211 10.1111/j.1348-0421.2012.00471.x

[28] Benoit MR, Klaus DM (2005) Can genetically modified *Escherichia coli* with neutral buoyancy induced by gas vesicles be used as an alternative method to clinorotation for microgravity studies?. Microbiology 151: 69–74. 15632426 10.1099/mic.0.27062-0

[29] Baker PW, Leff LG (2004) The effect of simulated microgravity on bacteria from the Mir space station. Microgravity Sci Technol 15: 35–41. 15773020 10.1007/BF02870950

[30] Klaus D, Simske S, Todd P, Stodieck L (1997) Investigation of space flight effects on *Escherichia coli* and a proposed model of underlying physical mechanisms. Microbiology 143: 449. 9043122 10.1099/00221287-143-2-449

[31] Bouloc P (1991) D’Ari (1991) *Escherichia coli* metabolism in space. J Gen Microbiol 137: 2839–2843. 1791437 10.1099/00221287-137-12-2839

[32] Berry BJ, Jenkins DG, Schuerger AC (2010) Effects of simulated mars conditions on the survival and growth of Escherichia coli and Serratia liquefaciens. Appl Environ microbiol 76: 2377–2386. 20154104 10.1128/AEM.02147-09 PMC2849189

[33] Yuan L, Hillman JD, Progulske-Fox A (2005) Microarray Analysis of Quorum-Sensing-Regulated Genes in *Porphyromonas gingivalis.* . Infect Immun 73: 4146–4154. 15972504 10.1128/IAI.73.7.4146-4154.2005 PMC1168601

[34] Dalman MR, Deeter A, Nimishaka G, Duan Z-H (2012) Fold change and p-value cutoffs significantly alter microarray interpretations. BMC Bioinformatics 13 Suppl 2 S11. 10.1186/1471-2105-13-S2-S11 PMC3305783 22536862

[35] Crabbe A, Schurr MJ, Monsieurs P, Morici L, Schurr J, et al (2011) Transcriptional and proteomic responses of *Pseudomonas aeruginosa* PA01 to spaceflight conditions involve Hfq regulation and reveal a role for oxygen. Appl Environ Microbiol 77: 1221–1230. 21169425 10.1128/AEM.01582-10 PMC3067220

[36] Jagessar KL, Jain C (2010) Functional and molecular analysis of *Escherichia coli* strains lacking multiple DEAD-box helicases. RNA 16: 1386–1392. 20484467 10.1261/rna.2015610 PMC2885687

[37] Spitzer ED, Jimenez-Billini HE, Weiss B (1988) 1-Alanine auxotrophy associated with dfp, a locus affecting DNA synthesis in *Escherichia coli* . J Baceriol 170: 872–876. 10.1128/jb.170.2.872-876.1988 PMC210735 3123465

[38] Wilson HR, Turnbough JRCL (1990) Role of the purine repressor in the regulation of pyrimidine gene expression in *Escherichia coli* K-12. J Bacteriol 172: 3208–3213. 1971621 10.1128/jb.172.6.3208-3213.1990 PMC209126

[39] Mittenhuber G (2001) Comparative genomics and evolution of genes encoding bacterial (p)ppGpp synthetases/hydrolases (the Rel, RelA and SpoT Proteins). J Mol Microbiol Biotechnol 3: 585–600. 11545276

[40] Mechold U, Cashel M, Steiner K, Gentry D, Malke H (1996) Functional analysis of a *relA/spoT* gene homolog from *Streptococcus equisimilis.* . J Bacteriol 178: 1401–1411. 8631718 10.1128/jb.178.5.1401-1411.1996 PMC177815

[41] Parekh BS, Hatfield GW (1997) Growth rate-related regulation of the *ilvGMEDA* Operon of *Escherichia coli* K-12 Is a consequence of the polar frame shift mutation in the *ilvG* gene of this strain. J Bacteriol 179: 2086–2088. 9068661 10.1128/jb.179.6.2086-2088.1997 PMC178939

[42] Ikenaka SK, Ramakrishnan G, Inouye M, Tsung K, Inouye M (1986) Regulation of the *ompC* gene of *Escherichia coli.* Involvement of three tandem promoters. J biologic chem 261: 9316–9320. 3013884

[43] Chen S, Wilson DB (1997) Construction and characterization of *Escherichia coli* genetically engineered for bioremediation of Hg21-contaminated environments. Appl Env Microbiol 63: 2442–2445. 9172366 10.1128/aem.63.6.2442-2445.1997 PMC168538

[44] Nagakub S, Nishino K, Hirata T, Yamaguchi A (2002) The putative response regulator BaeR stimulates multidrug resistance of *Escherichia coli* via a novel multidrug exporter system, MdtABC. J Bacteriol 184: 4161–4167. 12107133 10.1128/JB.184.15.4161-4167.2002 PMC135206

[45] Leys N, Hendrickx L, De Bover P, Baatout S, Mergeay M (2004) Space flight effects on bacterial physiology. J Biol Regul Homeost Agents. 18: 193–199. 15471227

[46] Nachin L, El Hassouni M, Loiseau L, Expert D, Barras F (2001) SoxR-dependent response to oxidative stress and virulence of *Erwinia chrysanthemi*: the key role of SufC, an orphan ABC ATPase. Mol Microbiol 39: 960–972. 11251816 10.1046/j.1365-2958.2001.02288.x

[47] Ranquet C, Gottesman S (2007) Translational regulation of the *Escherichia coli* stress factor RpoS: a role for SsrA and Lon J Bacteriol. 189: 4872–4879. 10.1128/JB.01838-06 PMC1913435 17449615

