# Supplementary material for: Effect of Simulated Microgravity on E. coli K12 MG1655 Growth and Gene Expression
Source: PLoS One. 2013 Mar 5;8(3):e57860. doi: 10.1371/journal.pone.0057860 (PMC3589462; doi:10.1371/journal.pone.0057860)
Supplement: Table S1 — Glycerol-induced up regulation of genes in Escherichia coli. DNA microarray analysis of E. coli grown in the presence of 10% glycerol showed up regulation of 103 genes with a fold change >1.5 (P<0.05). (DOCX) [file pone.0057860.s001.docx]

**Table S1.** Glycerol-induced upregulation of genes in *Escherichia coli*

| **Probe set ID** | **Fold change^*^** | **Gene** | **Gene function** |
| --- | --- | --- | --- |
| 1767961_s_at | 1.9 | *ahpC* | alkyl hydroperoxide reductase subunit C |
| 1761043_s_at | 1.7 | *aidB* | isovaleryl CoA dehydrogenase |
| 1766282_s_at | 1.8 | *ansB* | L-asparaginase II |
| 1761540_s_at | 1.7 | *arcA* | two-component response regulator |
| 1765363_s_at | 3.0 | *dhaL* | dihydroxyacetone kinase subunit DhaL |
| 1759621_s_at | 2.0 | *c2623* | fructose-bisphosphate aldolase |
| 1764617_s_at | 1.6 | *cadA* | lysine decarboxylase, induBHcible |
| 1759972_s_at | 2.3 | *cadB* | lysine/cadaverine antiporter |
| 1767087_s_at | 2.0 | *cbpA* | curved DNA-binding protein CbpA |
| 1761834_s_at | 1.7 | *cbpM* | modulator of CbpA co-chaperone |
| 1766292_s_at | 1.6 | *cfa* | cyclopropane fatty acyl phospholipid synthase |
| 1765588_s_at | 2.1 | *cydB* | cytochrome D ubiquinol oxidase subunit II |
| 1761551_s_at | 1.8 | *cysQ* | adenosine-3'(2'),5'-bisphosphate nucleotidase |
| 1760489_s_at | 1.5 | *dcuA* | anaerobic C4-dicarboxylate transporter |
| 1764747_s_at | 3.5 | *dhaK* | dihydroxyacetone kinase, PTS-dependent, |
| 1760957_s_at | 1.6 | *dhaM* | dihydroxyacetone kinase subunit M |
| 1764019_s_at | 1.5 | *dkgA* | 2,5-diketo-D-gluconate reductase A |
| 1760052_s_at | 2.0 | *hlpA* | periplasmic chaperone |
| 1768146_s_at | 1.7 | *ykfE* | C-lysozyme inhibitor |
| 1759740_s_at | 2.4 | *ybaS* | glutaminase |
| 1766093_s_at | 2.0 | *ybaT* | putative amino acid/amine transport protein |
| 1765453_s_at | 1.6 | *hyaA* | hydrogenase-1 small subunit |
| 1763696_s_at | 3.0 | *yciD* | outer membrane protein W |
| 1768165_s_at | 3.0 | *xasA* | acid sensitivity protein |
| 1760545_s_at | 3.9 | *gadB* | glutamate decarboxylase isozyme |
| 1759996_s_at | 1.5 | *nemA* | N-ethylmaleimide reductase |
| 1768914_s_at | 2.1 | *tar* | methyl-accepting chemotaxis protein II |
| 1762258_s_at | 1.8 | *ftnA* | ferritin |
| 1764397_s_at | 1.8 | *yedU* | chaperone protein HchA |
| 1767932_s_at | 1.7 | *gatD* | galactitol-1-phosphate dehydrogenase |
| 1763390_s_at | 1.7 | *ECs3038* | putative oxidoreductase |
| 1759264_s_at | 1.6 | *yeiA* | dihydropyrimidine dehydrogenase |
| 1767068_s_at | 2.6 | *glpB* | anaerobic glycerol-3-phosphate dehydrogenase subunit B |
| **Probe set ID** | **Fold change^*^** | **Gene** | **Gene function** |
| 1764370_s_at | 2.4 | *yfiD* | autonomous glycyl radical cofactor GrcA |
| 1765094_s_at | 1.7 | *ygfK* | putative selenate reductase subunit ygfK |
| 1768076_s_at | 2.4 | *yqhD* | putative oxidoreductase |
| 1768250_s_at | 1.5 | *uspB* | universal stress protein UspB |
| 1768748_s_at | 3.2 | *uspA* | universal stress protein A stress global response regulator |
| 1768627_s_at | 2.0 | *slp* | Outer membrane protein Slp precursor |
| 1765321_s_at | 4.5 | *hdeA* | acid-resistance protein |
| 1765786_s_at | 2.0 | *hdeD* | acid-resistance membrane protein |
| 1764077_s_at | 2.0 | *gadW* | putative ARAC-type regulatory protein |
| 1768498_s_at | 6.9 | *gadA* | glutamate decarboxylase isozyme |
| 1762126_s_at | 4.0 | *tnaL* | tryptophanase leader peptide |
| 1766722_s_at | 1.6 | *metE* | 5-methyltetrahydropteroyltriglutamate--homocysteine S-methyltransferase |
| 1764599_s_at | 1.8 | *udp* | uridine phosphorylase |
| 1762274_s_at | 2.5 | *uspD* | universal stress protein UspD |
| 1767690_s_at | 1.9 | *katG* | hydroperoxidase HPI(I) |
| 1764083_s_at | 2.8 | *hupA* | transcriptional regulator HU subunit alpha |
| 1759219_s_at | 1.5 | *frdC* | fumarate reductase subunit C |
| 1767636_s_at | 1.8 | *frdA* | fumarate reductase flavoprotein subunit |
| 1761937_s_at | 1.6 | *rnr* | exoribonuclease R |
| 1766425_s_at | 1.5 | *priB* | primosomal replication protein N |
| 1767098_s_at | 1.7 | *fimA* | major type 1 subunit fimbrin (pilin) |
| 1767463_s_at | 1.5 | *osmY* | periplasmic protein |
| 1767885_at | 3.4 | *fliC* | flagellar filament structural protein (flagellin) |
| 1766190_s_at | 1.5 | *glgC* | glucose-1-phosphate adenylyltransferase |
| 1768388_s_at | 2.8 | *glpD* | glycerol-3-phosphate dehydrogenase |
| 1766804_s_at | 1.6 | *glpK* | glycerol kinase |
| 1759829_s_at | 3.2 | *hdeB* | acid-resistance protein |
| 1763159_s_at | 2.0 | *ldcC* | lysine decarboxylase, constitutive |
| 1762979_s_at | 1.8 | *lysS* | lysyl-tRNA synthetase |
| 1766915_s_at | 1.7 | *mscL* | large-conductance mechanosensitive channel |
| 1760483_at | 2.2 | *narG* | nitrate reductase 1, alpha subunit |
| 1765159_s_at | 1.5 | *pgl* | 6-phosphogluconolactonase |
| 1765465_at | 1.7 | *phnL* | carbon-phosphorus lyase complex subunit |
| **Probe set ID** | **Fold change^*^** | **Gene** | **Gene function** |
| 1763376_at | 1.5 | *rluF* | 23S rRNA U2604 pseudouridine synthase |
| 1760419_at | 1.6 | *smg* | conserved protein |
| 1762381_s_at | 2.5 | *spf* | ncRNA |
| 1766768_s_at | 1.7 | *thrL* | thr operon leader peptide |
| 1762639_s_at | 1.7 | *tktB* | transketolase |
| 1761050_s_at | 1.9 | *tnaA* | tryptophanase |
| 1762367_s_at | 1.7 | *tpiA* | triosephosphate isomerase |
| 1766101_s_at | 3.0 | *wrbA* | TrpR binding protein WrbA |
| 1764790_at | 1.8 | *ybjP* | lipoprotein |
| 1765337_s_at | 1.7 | *ygeW* | aspartate/ornithine carbamoyltransferase family protein |
| 1767571_s_at | 1.7 | *ybaY* | hypothetical protein |
| 1768363_s_at | 2.5 | *ybdQ* | hypothetical protein |
| 1769106_s_at | 3.9 | *yccJ* | hypothetical protein |
| 1768740_s_at | 2.0 | *yoaC* | hypothetical protein |
| 1760888_s_at | 1.5 | *ECs2638* | hypothetical protein |
| 1762657_s_at | 1.8 | *yqaE* | hypothetical protein |
| 1765341_s_at | 2.4 | *yqjC* | hypothetical protein |
| 1764293_s_at | 2.2 | *yqjD* | hypothetical protein |
| 1762735_s_at | 1.6 | *yhhA* | hypothetical protein |
| 1768136_s_at | 1.5 | *yhiM* | hypothetical protein |
| 1762814_s_at | 1.6 | *yieF* | hypothetical protein |
| 1767716_s_at | 2.5 | *yiiS* | hypothetical protein |
| 1764349_s_at | 3.6 | *yjbQ* | hypothetical protein |
| 1763249_s_at | 1.9 | *yjbR* | hypothetical protein |
| 1762919_s_at | 1.5 | *yjcE* | hypothetical protein |
| 1769100_s_at | 1.7 | *yjdI* | hypothetical protein |
| 1760057_s_at | 1.5 | *yjeI* | hypothetical protein |
| 1764487_s_at | 1.5 | *yjgD* | hypothetical protein |
| 1760727_s_at | 2.3 | *ygaM* | hypothetical protein |
| 1763541_s_at | 1.7 | IG | intergenic region |
| 1768754_s_at | 1.6 | IG | intergenic region |
| 1764440_s_at | 2.0 | IG | intergenic region |
| 1759914_s_at | 1.7 | IG | intergenic region |
| 1763050_s_at | 1.9 | IG | intergenic region |
| 1765239_s_at | 1.6 | IG | intergenic region |
| 1768037_s_at | 2.2 | IG | intergenic region |
| 1762913_s_at | 1.7 | IG | intergenic region |

*Genes that showed fold change greater than 1.5 (P < 0.05)
