# Supplementary material for: Effect of Simulated Microgravity on E. coli K12 MG1655 Growth and Gene Expression
Source: PLoS One. 2013 Mar 5;8(3):e57860. doi: 10.1371/journal.pone.0057860 (PMC3589462; doi:10.1371/journal.pone.0057860)
Supplement: Table S2 — Glycerol-induced down regulation of genes in Escherichia coli . DNA microarray analysis of E. coli grown in the presence of 10% glycerol showed down regulation of 209 genes with a fold change >1.5 (P<0.05). (DOCX) [file pone.0057860.s002.docx]

**Table S2.** Glycerol-induced down regulation of genes in *Escherichia coli*

| **Probe set ID** | **Fold change^*^** | **Gene** | **Gene function** |
| --- | --- | --- | --- |
| 1763981_s_at | 1.8 | *aceA* | isocitrate lyase |
| 1761179_at | 4.8 | *aceB* | malate synthase |
| 1762343_s_at | 2.9 | *aceB* | malate synthase |
| 1765758_s_at | 3.0 | *aceK* | bifunctional isocitrate dehydrogenase kinase/phosphatase protein |
| 1764023_s_at | 1.8 | *aldA* | aldehyde dehydrogenase A |
| 1759685_s_at | 1.6 | *araF* | L-arabinose-binding periplasmic protein precursor |
| 1764514_s_at | 1.6 | *aroE* | quinate/shikimate dehydrogenase |
| 1768176_at | 1.6 | *arrQ* | qin prophage |
| 1759494_s_at | 1.7 | *ycfR* | biofilm, cell surface and signaling protein |
| 1767634_s_at | 1.6 | *dhaR* | DNA-binding transcriptional regulator DhaR |
| 1761434_s_at | 1.5 | *ydeT* | outer membrane usher protein fimD precursor |
| 1760530_s_at | 1.6 | *cedA* | cell division modulator |
| 1761593_s_at | 1.5 | *celC* | PTS system N,N'-diacetylchitobiose-specific transporter subunit IIA |
| 1766291_s_at | 1.5 | *clcB* | chloride channel, voltage-gated |
| 1760176_s_at | 1.6 | *yecO* | tRNA cmo(5)U34 methyltransferase, SAM-dependent |
| 1766086_s_at | 2.8 | *cobT* | nicotinate-nucleotide--dimethylbenzimidazole phosphoribosyltransferase |
| 1766210_at | 1.6 | *csgA* | cryptic curlin major subunit |
| 1762325_s_at | 2.0 | *csgB* | curlin minor subunit |
| 1762932_s_at | 1.9 | *cspB* | cold shock-like protein cspB |
| 1760599_s_at | 1.9 | *cspI* | cold shock-like protein cspI |
| 1761088_s_at | 1.5 | *cybB* | cytochrome b561 |
| 1759374_s_at | 1.5 | *cyoB* | cytochrome o ubiquinol oxidase subunit I |
| 1765526_s_at | 2.0 | *cysJ* | sulfite reductase subunit alpha |
| 1763056_s_at | 1.6 | *ddpA* | D-ala-D-a la transporter subunit |
| 1767620_s_at | 1.5 | *ddpC* | D-ala-D-ala transporter subunit |
| 1763357_s_at | 5.3 | *dnaK* | molecular chaperone DnaK |
| 1764562_s_at | 1.5 | *mhpT* | putative 3-hydroxyphenylpropionic transporter MhpT |
| 1762794_s_at | 1.6 | *ylaC* | inner membrane protein, DUF1449 family |
| 1761819_s_at | 1.5 | *htpG* | molecular chaperone HSP90 family |
| 1760290_s_at | 1.8 | *sucB* | dihydrolipoamide succinyltransferase |
| 1768954_s_at | 1.6 | *ycbL* | predicted metal-binding enzyme |
| 1763745_s_at | 1.6 | *ycbV* | putative fimbrial-like protein |
| 1765036_s_at | 1.6 | *hspQ* | heat shock protein HspQ |
| 1765839_s_at | 1.5 | *plsX* | putative glycerol-3-phosphate acyltransferase PlsX |
| 1767455_s_at | 1.6 | *purB* | adenylosuccinate lyase |
| **Probe set ID** | **Fold change^*^** | **Gene** | **Gene function** |
| 1761216_s_at | 3.0 | *nohB* | terminase small subunit |
| 1760637_s_at | 1.5 | *fadR* | fatty acid metabolism regulator |
| 1764969_s_at | 3.0 | *oppA* | oligopeptide transport periplasmic binding protein |
| 1763143_s_at | 1.7 | *topA* | DNA topoisomerase I |
| 1761544_s_at | 1.6 | *rnb* | exoribonuclease II |
| 1760255_s_at | 1.6 | *yciW* | putative oxidoreductase |
| 1769101_s_at | 3.6 | *pspA* | phage shock protein PspA |
| 1762530_s_at | 2.5 | *pspC* | DNA-binding transcriptional activator PspC |
| 1765796_s_at | 1.6 | *pspD* | peripheral inner membrane phage-shock protein |
| 1762511_s_at | 1.6 | *ynbB* | putative phosphatidate cytidiltransferase |
| 1762566_s_at | 1.5 | *ydcP* | putative collagenase |
| 1764131_s_at | 1.6 | *ydcS* | putative transport protein |
| 1768514_s_at | 1.6 | *nhoA* | N-hydroxyarylamine O-acetyltransferase |
| 1765054_s_at | 1.7 | *yddL* | putaive outer membrane porin protein |
| 1760891_s_at | 1.6 | *fdnI* | formate dehydrogenase-N subunit gamma |
| 1764150_s_at | 1.7 | *pqqL* | putative peptidase |
| 1768362_s_at | 1.5 | *ydeO* | transcriptional regulator YdeO |
| 1765383_s_at | 1.6 | *ydeP* | putative oxidoreductase |
| 1768967_s_at | 1.5 | *ydeR* | putative fimbrial-like protein |
| 1768257_s_at | 1.6 | *ydeV* | autoinducer-2 (AI-2) kinase |
| 1761744_s_at | 1.5 | *ydeY* | putative transport system permease protein |
| 1761065_s_at | 1.5 | *ydeZ* | putative transport system permease protein |
| 1767864_s_at | 1.5 | *yneB* | aldolase |
| 1763406_s_at | 1.6 | *yneH* | glutaminase |
| 1761440_s_at | 1.6 | *ynfL* | putative transcriptional regulator LYSR-type |
| 1763040_s_at | 1.6 | *mdtI* | multidrug efflux system protein MdtI |
| 1759134_s_at | 1.5 | *mdtJ* | multidrug efflux system protein MdtJ |
| 1768956_s_at | 1.5 | *rstA* | DNA-binding transcriptional regulator RstA |
| 1759546_s_at | 1.5 | *tus* | DNA replication terminus site-binding protein |
| 1767184_s_at | 2.0 | *fumA* | fumarase A |
| 1760033_s_at | 1.5 | *yniC* | 2-deoxyglucose-6-phosphatase deoxyglucose-6-phosphatase |
| 1761943_s_at | 1.6 | *ynjD* | putative ATP-binding component of a transport system |
| 1761643_s_at | 1.6 | *topB* | DNA topoisomerase III |
| 1764315_s_at | 1.6 | *ydjF* | putative DEOR-type transcriptional regulator |
| **Probe set ID** | **Fold change^*^** | **Gene** | **Gene function** |
| 1760010_s_at | 1.6 | *ydjK* | putative transport protein |
| 1763267_s_at | 1.6 | *yeaM* | putative ARAC-type regulatory protein |
| 1762151_s_at | 1.5 | *fliT* | flagellar biosynthesis protein FliT |
| 1768717_s_at | 1.5 | *yeeF* | predicted amino-acid transporter |
| 1762857_s_at | 1.5 | *lysP* | lysine transporter |
| 1764573_s_at | 1.5 | *yeiE* | putative DNA-binding transcriptional regulator |
| 1768233_s_at | 1.6 | *gyrA* | DNA gyrase subunit A |
| 1767449_s_at | 1.6 | *yqfA* | putative oxidoreductase |
| 1767563_s_at | 2.6 | *rpoA* | DNA-directed RNA polymerase subunit alpha |
| 1762768_s_at | 2.7 | *rpsK* | 30S ribosomal protein S11 |
| 1763295_s_at | 2.9 | *hokB* | small toxic polypeptide |
| 1763985_s_at | 1.6 | *ffs* | ncRNA |
| 1768518_s_at | 2.0 | *groEL* | chaperonin GroEL |
| 1763841_at | 2.0 | *hrpA* | predicted ATP-dependent helicase |
| 1765954_s_at | 1.9 | *insH* | IS5 transposase and trans- |
| 1766197_s_at | 2.0 | *mltD* | membrane-bound lytic murein transglycosylase D |
| 1762726_s_at | 1.6 | *mnmA* | tRNA-specific 2-thiouridylase MnmA |
| 1762123_s_at | 1.7 | *narU* | nitrite extrusion protein 2 |
| 1761726_s_at | 2.6 | *oppB* | oligopeptide transporter permease |
| 1765783_s_at | 2.0 | *oppD* | oligopeptide transporter ATP-binding component |
| 1767860_at | 1.6 | *paaA* | ring 1,2-phenylacetyl-CoA epoxidase subunit |
| 1764442_at | 1.6 | *paaE* | ring 1,2-phenylacetyl-CoA epoxidase, NAD(P)H oxidoreductase component |
| 1765606_at | 1.6 | *paaI* | thioesterase, most active with ring-hydroxylated phenylacetyl-coenzyme A thioesters |
| 1763270_at | 1.6 | *paaK* | phenylacetyl-CoA ligase |
| 1759599_s_at | 1.6 | *potA* | putrescine/spermidine ABC transporter ATPase protein |
| 1760103_s_at | 1.6 | *pphA* | serine/threonine protein phosphatase 1 |
| 1767618_s_at | 1.7 | *prfA* | peptide chain release factor 1 |
| 1768435_s_at | 1.7 | *pspB* | phage shock protein B |
| 1766134_at | 1.6 | *relA* | (p)ppGpp synthetase I/GTP pyrophosphokinase |
| 1760110_at | 1.8 | *relB* | bifunctional antitoxin of the RelE-RelB toxin-antitoxin system |
| 1761308_at | 1.6 | *rem* | qin prophage |
| 1767883_s_at | 2.7 | *rimM* | 16S rRNA-processing protein RimM |
| 1766400_s_at | 2.1 | *rplF* | 50S ribosomal protein L6 |
| 1760773_s_at | 2.5 | *rplN* | 50S ribosomal protein L14 |
| 1764794_s_at | 1.9 | *rplO* | 50S ribosomal protein L15 |
| **Probe set ID** | **Fold change^*^** | **Gene** | **Gene function** |
| 1765175_s_at | 1.5 | *rpsP* | 30S ribosomal protein S16 |
| 1768787_s_at | 1.6 | *rspB* | putative dehydrogenase |
| 1765303_at | 1.6 | *rzpQ* | Rz-like equivalent, Qin prophage |
| 1769260_s_at | 1.7 | *sdhB* | succinate dehydrogenase iron-sulfur subunit |
| 1767040_s_at | 1.8 | *sdhD* | succinate dehydrogenase cytochrome b556 small membrane subunit |
| 1759132_at | 1.7 | *sokB* | ncRNA |
| 1760295_s_at | 1.5 | *sppA* | protease 4 |
| 1766868_s_at | 1.5 | *sucA* | 2-oxoglutarate dehydrogenase E1 component |
| 1769042_s_at | 2.0 | *sucC* | succinyl-CoA synthetase subunit beta |
| 1760848_s_at | 1.6 | *tmk* | thymidylate kinase |
| 1764937_s_at | 1.6 | *torD* | chaperone protein TorD |
| 1764493_s_at | 2.0 | *trmD* | tRNA (guanine-N(1)-)-methyltransferase |
| 1760194_s_at | 2.5 | *wzxC* | colanic acid exporter |
| 1764008_s_at | 1.6 | *ybeQ* | hypothetical protein |
| 1765399_at | 1.7 | *ycaI* | inner membrane protein, ComEC family of competence proteins |
| 1762340_at | 1.5 | *ydbA* | pseudo |
| 1767247_at | 1.6 | *ydfE* | pseudo |
| 1763158_at | 1.5 | *ydfU* | qin prophage |
| 1767166_at | 1.5 | *ydfX* | pseudo |
| 1769219_at | 1.6 | *yedS* | pseudo |
| 1764941_at | 1.7 | *yehH* | pseudo |
| 1765532_at | 1.5 | *yfcS* | predicted periplasmic pilus chaperone |
| 1767447_s_at | 2.0 | *yhcE* | pseudo |
| 1765949_s_at | 1.7 | *znuA* | high-affinity zinc transporter periplasmic component |
| 1760502_s_at | 1.6 | *znuB* | high-affinity zinc transporter membrane component |
| 1759812_s_at | 1.5 | *znuC* | high-affinity zinc transporter ATPase |
| 1760598_s_at | 1.9 | *Z2403* | hypothetical protein |
| 1761606_s_at | 1.5 | *yedI* | hypothetical protein |
| 1768215_s_at | 1.6 | *c1929* | hypothetical protein |
| 1762061_s_at | 1.7 | *ybiJ* | hypothetical protein |
| 1764771_s_at | 1.6 | *ycbK* | hypothetical protein |
| 1759715_s_at | 1.5 | *yccA* | hypothetical protein |
| 1764800_s_at | 1.5 | *ycdO* | hypothetical protein |
| 1765329_s_at | 2.3 | *ydfD* | hypothetical protein |
| 1768270_s_at | 1.6 | *ycjX* | hypothetical protein |
| 1760169_s_at | 1.5 | *ydaQ* | hypothetical protein |
| 1767994_s_at | 1.7 | *ynbD* | hypothetical protein |
| 1762519_s_at | 1.7 | *ECs2030* | hypothetical protein |
| 1760300_s_at | 1.5 | *yncH* | hypothetical protein |
| **Probe set ID** | **Fold change^*^** | **Gene** | **Gene function** |
| 1760165_s_at | 1.5 | *yneF* | hypothetical protein |
| 1762245_s_at | 1.7 | *ydeH* | hypothetical protein |
| 1769059_s_at | 1.5 | *ynfC* | hypothetical protein |
| 1765419_s_at | 1.9 | *ECs2526* | hypothetical protein |
| 1768183_s_at | 1.5 | *yebO* | hypothetical protein |
| 1767917_s_at | 1.6 | *yebE* | hypothetical protein |
| 1759130_s_at | 1.7 | *ydhL* | hypothetical protein |
| 1761110_s_at | 1.8 | *ydhZ* | hypothetical protein |
| 1760257_s_at | 1.5 | *ppsR* | hypothetical protein |
| 1764585_at | 1.5 | *-* | unknown |
| 1764779_at | 1.6 | *-* | unknown |
| 1766272_s_at | 1.6 | IG | intergenic region |
| 1759377_s_at | 1.7 | IG | intergenic region |
| 1769180_s_at | 1.6 | IG | intergenic region |
| 1763183_s_at | 1.7 | IG | intergenic region |
| 1766595_s_at | 1.5 | IG | intergenic region |
| 1767517_s_at | 1.7 | IG | intergenic region |
| 1765268_s_at | 1.7 | IG | intergenic region |
| 1762784_s_at | 1.7 | IG | intergenic region |
| 1762102_s_at | 1.6 | IG | intergenic region |
| 1761933_s_at | 2.0 | IG | intergenic region |
| 1760857_s_at | 1.6 | IG | intergenic region |
| 1759623_s_at | 1.5 | IG | intergenic region |
| 1762043_s_at | 1.6 | IG | intergenic region |
| 1764389_s_at | 1.5 | IG | intergenic region |
| 1759370_s_at | 1.5 | IG | intergenic region |
| 1764282_s_at | 1.5 | IG | intergenic region |
| 1767526_s_at | 1.5 | IG | intergenic region |
| 1763779_s_at | 1.5 | IG | intergenic region |
| 1760035_s_at | 1.6 | IG | intergenic region |
| 1765865_s_at | 1.5 | IG | intergenic region |
| 1769161_s_at | 1.6 | IG | intergenic region |
| 1763393_s_at | 1.6 | IG | intergenic region |
| 1763179_s_at | 1.6 | IG | intergenic region |
| 1759577_s_at | 1.5 | IG | intergenic region |
| 1768016_s_at | 1.7 | IG | intergenic region |
| 1767198_s_at | 1.6 | IG | intergenic region |
| 1763395_s_at | 1.9 | IG | intergenic region |
| 1769034_s_at | 2.3 | IG | intergenic region |
| 1768367_s_at | 1.6 | IG | intergenic region |
| 1767722_s_at | 1.6 | IG | intergenic region |
| 1766159_s_at | 1.7 | IG | intergenic region |
| 1760296_s_at | 1.6 | IG | intergenic region |
| 1763854_s_at | 1.6 | IG | intergenic region |
| **Probe set ID** | **Fold change^*^** | **Gene** | **Gene function** |
| 1762302_s_at | 1.5 | IG | intergenic region |
| 1760520_s_at | 1.5 | IG | intergenic region |
| 1759210_s_at | 1.6 | IG | intergenic region |
| 1763955_s_at | 1.5 | IG | intergenic region |
| 1768588_s_at | 1.5 | IG | intergenic region |
| 1764685_s_at | 1.7 | IG | intergenic region |
| 1761883_s_at | 1.6 | IG | intergenic region |
| 1764821_s_at | 1.9 | IG | intergenic region |
| 1768692_s_at | 1.5 | IG | intergenic region |
| 1759679_s_at | 2.0 | IG | intergenic region |
| 1759846_s_at | 1.5 | IG | intergenic region |
| 1766143_s_at | 1.5 | IG | intergenic region |
| 1761336_s_at | 1.5 | IG | intergenic region |
| 1762591_s_at | 1.6 | IG | intergenic region |
| 1760503_s_at | 1.5 | IG | intergenic region |
| 1769272_s_at | 1.9 | IG | intergenic region |
| 1760059_s_at | 1.7 | IG | intergenic region |
| 1761124_s_at | 1.6 | IG | intergenic region |
| 1761725_s_at | 1.5 | IG | intergenic region |
| 1761720_s_at | 1.5 | IG | intergenic region |
| 1762804_s_at | 1.6 | IG | intergenic region |
| 1768829_s_at | 1.5 | IG | intergenic region |

*Genes that showed fold change greater than 1.5 (P < 0.05)
